# “*You can't even ask a question about your child*”: Examining experiences of parents or caregivers during hospitalization of their sick young children in Kenya: A qualitative study

**DOI:** 10.3389/frhs.2022.947334

**Published:** 2022-10-03

**Authors:** Chantalle Okondo, Charity Ndwiga, Pooja Sripad, Timothy Abuya, Charlotte E. Warren

**Affiliations:** ^1^Population Council, Nairobi, Kenya; ^2^Population Council, Washington, DC, United States

**Keywords:** Kenya, parents, caregivers, sick young children, health services, quality of care

## Abstract

**Background:**

Globally, about 5.2 million children under the age of five died in 2019, and more than half of those deaths occurred in Sub-Saharan Africa. In almost every death of a sick child, there is a parent/caregiver seeking health services for their child. This study sought to understand the experiences of care for parents/caregivers (caregivers) as they navigate the hospital system with the aim of identifying opportunities to improve service delivery and child health outcomes.

**Methods:**

Qualitative data were collected from five hospitals in Kenya: three in Nairobi County and two in Bungoma County. Twenty-five in-depth interviews with caregivers (couples and single women) of young children 0–24 months old, 17 focus group discussions with women and men, and 64 institutional ethnographic observations were completed. Data were analyzed by initial annotation of transcripts and field materials, followed by open coding and thematic analysis using Nvivo 12 software. Summary themes were used to compare experiences between female and male caregivers, their child's age group, and study sites.

**Results:**

Caregivers faced complex processes of care while seeking health services for their sick young children. Three overarching themes emerged with some variability across female and male caregiver perspectives: (1) *Navigating structural issues*: long wait times, confusing payment mechanisms, overcrowding, unhygienic conditions, and strict visitation policies; (2) *Interactions with providers*: positive experiences, including providers showing empathy and concern, and negative experiences of harsh language, neglect, lack of privacy, discounting caregiver perspectives, and not involving men; Limited communication between caregivers and providers on child's diagnosis, treatment, and progress and lack of communication specifically between male caregivers and providers; and (3) *Limited emotional support* for both caregivers during difficult diagnosis or bereavement.

**Conclusions:**

To improve experiences, interventions, programs, and policies need to focus on good provider-caregiver partnerships; enhancing opportunities for male engagement, such as supportive visiting hours; effective communication between caregivers and providers; access to adequate emotional support; and an enabling hospital environment.

## Introduction

Improving the quality of newborn and pediatric care remains central to preventing and reducing neonatal and young child mortality. Globally, 2.4 million children died in the first month of life and 5.2 million children under the age of five died in 2019 (1). Some interventions to improve child survival have targeted pregnancy, childbirth, the first weeks of life, small and sick newborns, breastfeeding, infant and young child feeding, and immunizations, among others (2–5). However, there are limited interventions to improve parents' or caregiver's perceived quality of maternal, newborn, and child health (MNCH) services, which can influence care seeking behaviors (3, 6) as well as limited interventions that seek to improve experiences of care. In Kenya, despite policies and strategies that have been implemented to improve child health, such as removal of user fees and the integrated management of newborn and childhood illnesses (7), barriers, such as the experience of harsh treatment from providers and hidden costs, prevent users from seeking care and limited interventions that focus on experience of care (8). The World Health Organization (WHO)'s maternal and newborn health and pediatric care frameworks call for attention to eight domains to improve quality of care (9, 10). Under the “Experience of Care” category, they highlight the need for: (1) effective communication with and meaningful participation of caregivers; (2) respect, protection, and fulfillment of children's rights; and (3) emotional and psychological support of children and their families (10). Many other guidelines emphasize family centered approaches to pediatric health care and responsive caregiving as part of early childhood development interventions (11, 12). As critical actors in navigating the facility environment and promoting their child's health, parents/caregivers need to feel heard as part of building mutual trust with the healthcare professionals. Their confidence in the health care system will be important for continuity of care once children are released from the hospital. Hereafter, we will use the term “caregivers” to refer to parents, aunts, uncles, grandmothers and/or legal guardians of sick young children 0–24 months.

In Kenya, mistreatment of women during childbirth has been well-documented and reported (13–15). Bohren et al. developed a global typology for mistreatment of women during childbirth (16), which Sacks expanded upon to define disrespect and abuse of newborns (17). Some of the mistreatment that caregivers experience includes providers blaming them for poor neonatal outcomes, denial of or threatening to deny postnatal care for those who give birth at home, and unsupportive care (17). A study in rural Rwanda found both positive and negative experiences of care for caregivers with newborns admitted to the neonatal care unit such as having full access and being part of their newborn's care; stress seeing their newborns in the newborn intensive care unit; or mixed provider attitudes, with some healthcare providers being encouraging while others being rude and showing no empathy (18). Caregivers in Kenya and Uganda who experienced stillbirths also reported poor interactions with health workers, lack of support and not being treated with compassion (19). Some of the drivers of newborn mistreatment include high patient workload in public health facilities, insufficient providers and limited infrastructure which may exacerbate poor service delivery (20). Previous research has shown that nurses who work in high-stress, low-resource environments with minimal managerial oversight may develop coping strategies, like closely monitoring high risk patients and rationing resources, facilitating limited direct emotional exposure to patients (21). There is a paucity of research in low- and middle-income countries (LMICs) on the experience of caregivers of sick young children, and specifically their needs, challenges, and opportunities in interacting with the health care system. For this paper we use the term “sick young children” to refer to newborns, infants, and young children aged 0–24 months receiving inpatient and outpatient services at a hospital. We explore the caregiver's experiences of care while receiving health services for their sick young children in five hospitals in Kenya.

## Methods

### Study design

The study adopted a cross-sectional, qualitative study with an ethnographic approach to understand caregiver's experiences from their perspectives, the subjective meanings they attach to these experiences, and to gain a more holistic picture of the hospital settings. The data presented in this paper comes from and informed a larger implementation research effort to develop and test strategies or models for provision of nurturing care and promotion of family engagement, communication, and respect for sick young children aged 0–24 months receiving hospital services in resource-constrained settings. A mix of qualitative data collection methods were used, including in-depth interviews (IDIs), focus group discussion (FGDs), and ethnographic observations, to understand the challenges caregivers faced and opportunities for the health system to provide support to caregivers during in-patient and outpatient care (22).

### Study context

This study was implemented in five hospitals in two counties (Bungoma and Nairobi) in Kenya. The selection of study facilities was done in consultation with the county health management teams and the national Ministry of Health. In Bungoma County, data were collected from two public referral hospitals, and in Nairobi County, data were collected from three facilities: one large public maternity hospital, one large tertiary public hospital, and a faith-based hospital. Bungoma County, located in Western Kenya, represents a rural area, where about half of the population (48%) is below 15 years and with a total fertility rate of 5.0, which is higher than the national average of 3.9 (23). In contrast, Nairobi County, which also serves as the capital, represents an urban area where 30% of the population are below the age of 15 years with a total fertility rate of 2.7 (23). Both counties have neonatal (0–4 weeks) mortality rates higher than the national average of 22 per 1,000 live births: 33/1,000 live births in Bungoma and 39/1,000 live births in Nairobi, which is also the highest in Kenya. Infant (<12 months) mortality rates in the two counties are also higher than the national estimates of 39/1,000 live births: Bungoma 97/1,000 live births and Nairobi 60/1,000 live births (23).

#### Study participants

The study participants were caregivers, which included mothers, fathers, family members, or legal guardians of sick young children aged between 0 and 24 months who sought inpatient or outpatient health care services in the five selected hospitals. The study team initially visited selected facilities to introduce the study to facility managers and department heads. Purposive sampling was used to identify study participants. Caregivers were approached face-to-face by the research team with assistance from hospital staff. We also used patient records to identify caregivers with children in the target age group (0–24 months) and hospital staff introduced the research assistants to the caregivers. Enumerators were trained on how to interact, build trust and be compassionate with parents (they were also advised not to approach parents who were visibly stressed, uncomfortable or vulnerable and stop any interviews if parents did become distressed and refer cases). All interviews were conducted in a private room away from hospital staff or in a place of their choosing.

#### Data collection

Data collection was conducted in November and December 2019 (see Table 1 for the study activities completed). Research assistants with experience in qualitative methods and a clinical and/or social science background were trained by the study team on the protocol, research ethics, and conducting IDIs, FGDs, and ethnographies. Semi-structured interview guides were prepared in English, translated into Kiswahili, and pilot tested with research assistants. The interviews lasted between 60 and 90 min and were audio-recorded with the signed informed consent of the participants.

**Table 1:** Study activities.

**Methods (total number completed)**	**Nairobi**		**Bungoma**	
	**F**	**M**	**F**	**M**
IDIs with both caregivers (male and female) (23)	11	11	12	12
IDIs with single caregivers (female) (2)	1	0	1	0
FGDs with female caregivers (8)	46	0	23	0
FGDs with male partners (fathers) (4)	0	11	0	14
**Total number of participants**	**58**	**22**	**36**	**26**
**Study Method**	**Nairobi**	**Bungoma**
Ethnographic observations	33	31

#### Interviews with single caregivers and couples

Caregivers whose sick young children were receiving care in the study facilities were recruited from various departments where children were being treated. We conducted a total of 25 IDIs, 23 IDIs with couples and two with single women. Most caregivers interviewed received inpatient services for their sick child, while seven caregivers (couples) that initially visited the facility for outpatient services resulted in admissions. Deliberate efforts were made to include caregivers who lost their babies to explore problems and barriers linked to the deaths of infants. Caregiver couples of sick young children identified in the facilities were invited to participate in a joint interview in a comfortable private location (the facilitator asked questions directly to the couple and encouraged both caregivers to share their views/experiences). Topics for all respondents included their experience of hospitalization, interactions with providers and the health system, their understanding and experience of respectful care for young children, and the underlying drivers of negative experiences. In addition, we explored with caregivers how providers could better partner with them to improve care and management of sick young children, including emotional and social support needed to manage the challenges associated with being a caregiver of sick young children (see Supplementary materials for interview guides).

#### Focus group discussions with caregivers

In each of the two counties, we conducted separate FGDs with women and men with young children recently hospitalized for treatment or born preterm and/or unwell (Table 1). We conducted a total of 12 FGDs (seven in Nairobi and five in Bungoma). One set of FGDs was conducted with women who gave birth in a hospital and had newborn babies, another set was conducted with women with children <2 years who had been recently hospitalized, and the third category was with a group of women with preterm births. This third group was included to specifically explore the support they might need when managing infants born early. A fourth group included husbands of the women with hospitalized young children. It was challenging to recruit men to participate in FGDs due to lack of time, they either had to go back to work or had competing tasks such as running errands. The FGDs focused on understanding the needs of women and men while managing children who are sick, manifestations of mistreatment of newborns and how to mitigate them, as well as caregiver experiences interacting with health providers. Other topics included perceptions of quality of health services provided, nature of interactions between caregivers and healthcare providers, did they feel they were treated with respect and dignity; did providers use appropriate language and were their opinions respected and included in caregiving as well as the underlying drivers of negative experiences (see Supplementary materials for FGD guide). Each FGD was facilitated by two research assistants: one facilitator and one note-taker in locations that were convenient to most of the participants. When necessary, FGDs were paused to allow time for breastfeeding or giving urgent attention to sick young children.

#### Ethnographic observations

We conducted ethnographic observations to understand how workflows and caregiver-provider interactions manifest in various departments where children were being treated. For each of the study facilities, 64 non-participatory observations were conducted over ten consecutive days, including weekends. We observed the following departments: maternity units, postnatal wards, newborn units, kangaroo mother care rooms, inpatient pediatric wards, and outpatient departments, including MCH outpatient and specialized clinics. Study teams sought approvals from the heads of the facilities, who were informed of the purpose of the observations and assured of confidentiality. Thereafter, the departmental heads were also approached and were requested to allow the team to conduct observations and record all relevant information using a narrative template that captured how providers interact with young children, caregivers, families, and among themselves.

An open-ended, structured template guided notetaking by a pair of research assistants (one with a clinical background and another with social science ethnography skills) (see Supplementary materials). The template focused on understanding the organizational culture and the dynamics of care processes for various services provided to sick young children through examining client flow, interactions, and bottlenecks of managing sick young children. This helped the study team to identify potential opportunities for caregivers to partner with providers as part of routine service delivery for sick young children. We also tracked the process of care for the average patient, from registration to discharge, and plans for follow up care, documenting all service points and care given in each location. Finally, we documented observations of mistreatment that occurred during the data collection period.

#### Analysis

All data were submitted to a centralized location for synthesis and storage. All audio-recorded interviews were transcribed verbatim and Kiswahili transcripts were translated into English. Thematic analysis was conducted with the initial annotation of transcripts, field notes, and observations. An initial coding framework was developed by three members of the research team and refined through cross checking of codes on an initial subset of transcripts (see Supplementary materials for the codebook). Transcripts were exported into Nvivo 12 (QSR International) software with facility names redacted. Three members of the team coded the data using open coding and progressive categorization of issues. The coded data then went through a process of charting to summarize key themes and concepts including references and quotations. Charts enabled the team to compare themes across sites, gender, and age category of sick young children, as well as to explore similarities and differences in caregiver experiences. We grouped the data for caregivers of young infants 0–59 days separately from caregivers of young children 60 days to 24 months, mainly because these children were treated in different units of the hospitals (e.g., newborn units and pediatric wards). The data from the observations was used to augment emerging themes from the interviews and focus group discussions with a focus on age of the child and location of the facility.

## Results

A total of ninety-four women and forty-eight men participated in the study (Table 1). We present caregiver perspectives from the moment they visit the health facility, the admission processes up until discharge around three major emergent themes: navigating hospital environment; interactions and communications with providers; and emotional support while seeking care for their sick young children.

### Navigating hospital environment

#### Hospital environment

**Figure 1:**
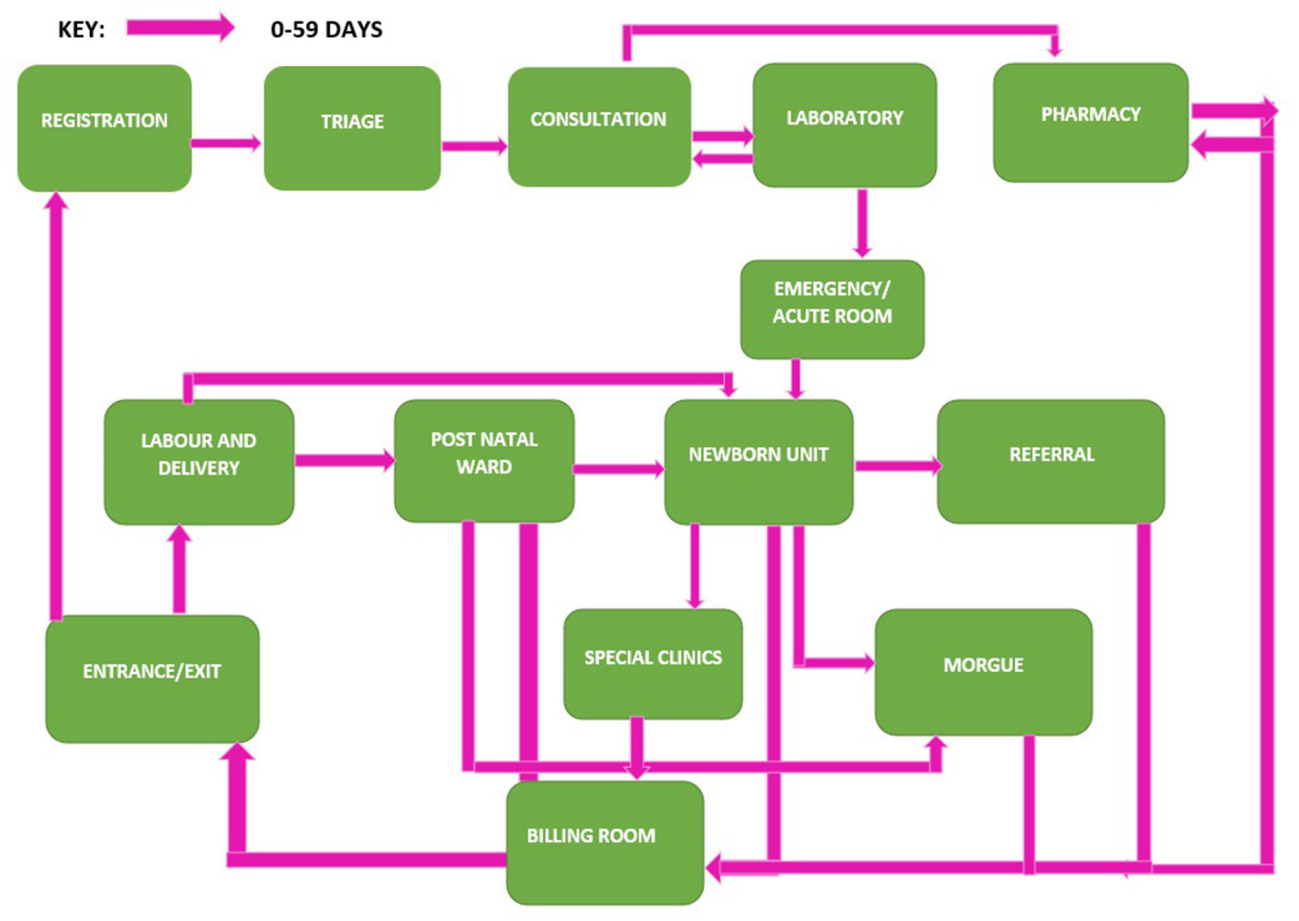
Client flow journey for caregiver of young children 0–59 days.

**Figure 2:**
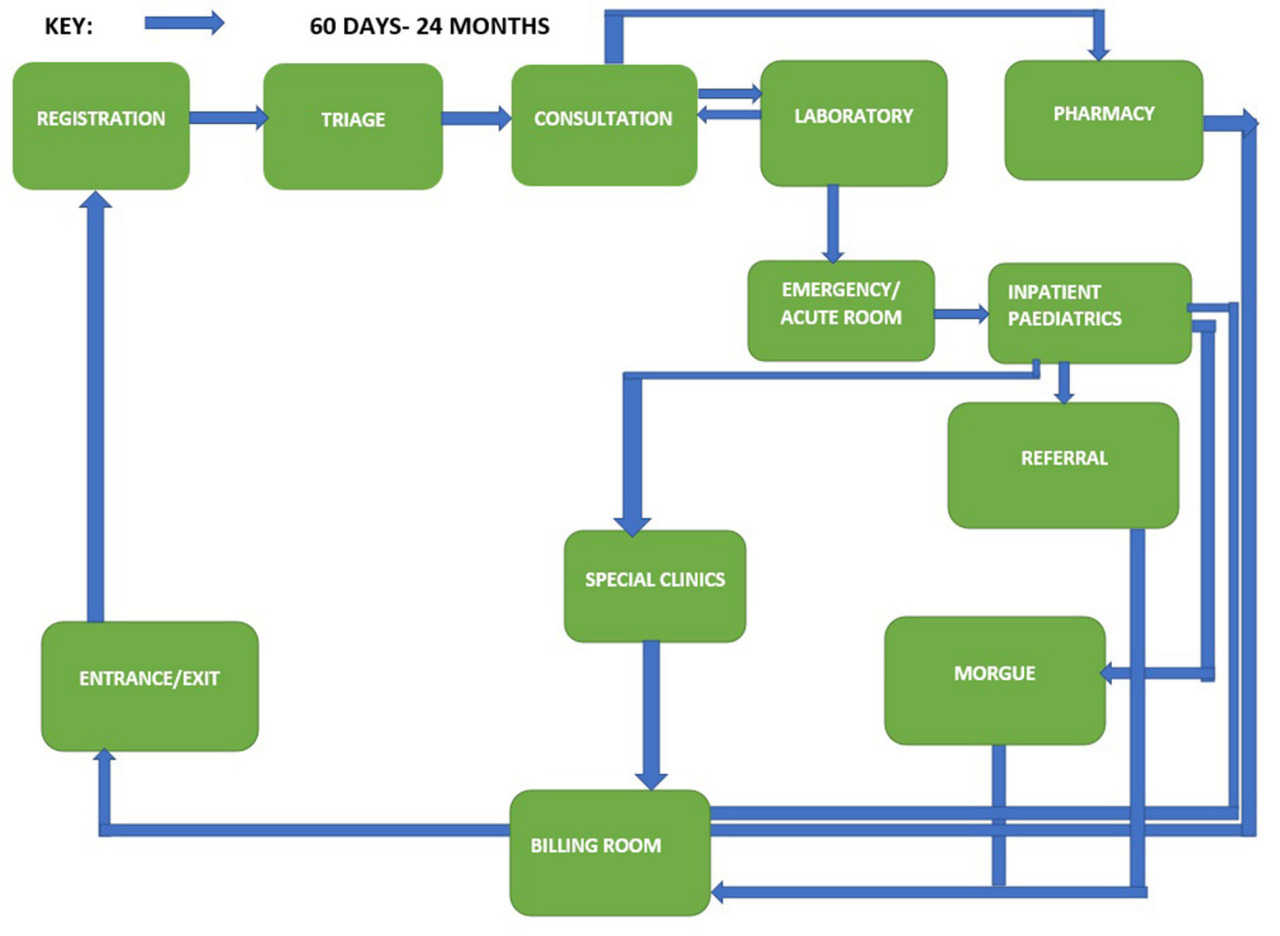
Client flow journey for caregiver of young children 60 days to 24 months.

We first present experiences of how caregivers navigate the physical spaces where care is given to children, we triangulate data from the interviews and focus group discussion with the observations to demonstrate similarities and differences between caregivers and facilities. Prior to admission, sick children and their caregivers visit at least five service points, which include registration, triage, consultation, laboratory and or radiology and emergency/acute room (see Figures 1, 2 for the client journey).^1^ In many cases we observed this led to delay in initiation of services, however in the faith-based facility most caregivers appreciated that there was a triage section and clinic designated for children therefore they did not have to wait long lines with other clients. During admissions in public facilities complex processes of care, shortages of staff at night and during the weekend resulted in waiting for laboratory results, lengthy queues, and delayed services. This was also reported in the interviews:

“*When I told the nurse the baby is feeling bad, she tells me to wait and remove the baby's clothes because the temperature was high. After a long wait, she sent me to the lab, after coming back with the results, she sent for the file… coming back she tells me again to wait, by the time I was admitted it was 3:00am”* FGD Women with young children (0–59 days)

Poor signage and a lack of orientation to the hospital environment made it challenging for caregivers to navigate the hospital system while receiving services. For example, in one hospital setting a client was observed being asked by the clinician to take the child for an X-ray; and when she asked the clinician where the radiology department was, she was told to get out and ask the security guards in the corridors for directions.

For inpatient postnatal women, it was challenging navigating distances between the postnatal ward and newborn units which were isolated from other wards for infection prevention and control purposes. During observations we found, restricted movement and rules around clothing in the newborn units compounded issues when nursing mothers were required to feed their newborns every 2–3 h. Mothers had to wear hospital gowns that were torn, too short or had loose buttons/fasteners that did not provide much coverage and warmth during cold nights. In one facility, the newborn unit was located at the end of a hallway that required nursing mothers dressed in hospital gowns to pass public waiting benches for men and women; mothers' visible discomfort was observed in response to people's stares. Once in the newborn unit, due to lack of space, they had to take turns entering the newborn unit and often felt rushed. One mother who had twins shared that it would be difficult to get both babies to wake up and feed within the specified time given by the nurses. Data from the interviews supported these observations:

“*Inside that room, there is a bench that can hold five mothers while seated, and for the rest they can't hold the baby for one hour while standing, so they just put them on their chest for a while and then leave. According to the seats and the space inside we can't all fit in that room at the same time, so you must wait for the others to finish before you go in,”* IDI, Couple with a young infant (0–59 days)

Unhygienic conditions were also experienced, where women reported being provided with soiled linen to lie on or being placed near the washrooms, which emitted a terrible stench due to poor cleaning; these experiences were “dehumanizing,” especially while nursing their newborns.

“*We just sleep on the floor, there is nothing to do because the beds are few and they have to prioritize the ones coming from theatre to sleep on the beds. So, we have to free up beds for other mothers, then we wait for long and later decide to sleep on the floor because of lack of space*.” IDI Couple with a young infant(0–59 days)

Observation data also established that women were subjected to crowded conditions within the postnatal and pediatric wards, such as sharing beds with their children, having to stand for long durations of time, or sitting on the floor. Structural features around care characterized by overcrowding and unhygienic conditions were mainly experienced by mothers in the public facilities and observed by the study team while in the faith-based facility caregivers' main concerns were around the cold temperature of the wards and feeling worried their babies were under dressed and not protected from the cold.

“*It was 9 pm and I was asked to wait for results, the baby had a fever and I asked if we could go to the ward because it was cold in the room, and I was also feeling cold but they told me I can't go without the results, I asked what about the windows and if we could close them, but because someone could come and maybe they are coughing this might lead to more infection”* FGD Women with young children (60 days to 24 months)

#### Hospital policies and processes

Another aspect of hospital environment is navigating hospital policies and processes. Data from the ethnographic observations established that in the faith-based facility, caregivers seeking services from outpatient pediatric departments were being turned away until they visited the billings office (on a different floor or section) and paid their registration fees; once paid, they had to line up again. Across all facilities, the payment process at several points for laboratory tests, X-rays, pharmacy, and at discharge made caregivers make several trips around the hospital and sometimes they got lost along the way. Delayed services were also observed where staff would be on prolonged tea and lunch breaks or caregivers in the outpatient pediatric department would wait 30–40 min after opening time to receive care for their sick children. Data from the interviews corroborate these observations:

“*You might go there (to the hospital) and the baby is in serious condition, then they (nurses) tell you to just queue. So you get more worried when will they attend to my baby. That state really offends me*.” IDI Couple with a young child (60 days to 24 months)

During inpatient care, limited visiting hours meant working men were not able to come to the hospital before 5 pm. Men would have to rely on support staff to help their wives/partners access services, such as being served food first or getting assigned a clean bed. Paying for bribes to access services was commonly reported, especially during discharge:

“*We as men we don't have time because you have to work to find money for discharge and what will she (my wife) eat… so by the time I get here its already late sometimes I can come at 9pm … I have to give the security guard Kshs 100 to get my wife food.”* FGD, Men

“*If the discharge process can be synchronized to one place, it will be very smooth. There was a time I saw the students asking for money around Kshs 200 so that they can help us through all these discharge processes.”* FGD, Men

### Caregiver interactions and communication with providers

This section describes female and male caregiver perspectives as it pertains to their interaction with health providers in the facility including how providers communicate with caregivers.

#### Perspectives from female caregivers

Along the journey of their children receiving inpatient or outpatient care, some female caregivers described having positive interactions with providers, such as being greeted nicely, some providers showed concern by asking mothers how they were doing or checking on them regularly. Women often singled out specific providers who were approachable or were sympathetic.

“*Sometimes, they (provider) ask you what you are thinking about. They encourage us to place our thoughts with our babies and not think of other things. They comfort us that the babies will get healed, and we will go home.”* FGD, Women with young infants (0–59 days)

“*In the wards the nurse there was good… I told her the baby's legs were so cold and she came and encouraged me to keep the baby warm. She was very concerned. She even came back the next day in the afternoon and cracked jokes on how I was crying the previous day when the baby was too sick. She made me laugh. I felt good and encouraged.”* IDI, Couple with a young child (60 days to 24 months)

These positive experiences of interactions were also documented from the ethnographic observations for example in one facility, mothers were seen to be talking/asking/clarifying issues with providers who appeared very friendly to the mothers. At one point, a nurse was informed by another provider that there was a mother who was not able to breastfeed, however they did not have her name. The nurse would proceed to visit each cubicle asking the mothers who among them was not able to breastfeed and observing those with babies on their breasts until they found her. Once the mother was identified the nurse politely corrected her on how she had positioned her baby on the bed, before going ahead to guide her on how to breastfeed her child.

In the pediatric ward there was a mother whose 1-year-old child had difficulty in breathing, and it was established that he had a cyst in his throat and an operation was required. The child was taken to theater for the procedure at 9:00 a.m. in the company of his grandmother. By 5:00 p.m. the mother was visibly disturbed and worried, she would cry at some instances as she waited since she had not received any information of his condition. The nurse kept assuring her that the child was okay, when he was later brought back, she was notified that the procedure had been successful.

Unfortunately for many women, negative experiences around interactions with providers impacted their confidence in the care their sick child received. In interviews, female caregivers commonly experienced harsh language from providers, which they described as occurring when the provider did not think the woman was handling the infant well or maintaining proper hygiene. Women reported being shouted at when asking for help in handling sick or fragile newborns or receiving rude responses when asking why tests were being done. Similarly, a doctor in a postnatal ward was observed verbally abusing a woman and accusing her of refusing to breastfeed her baby, this was done in the presence of other caregivers. However, it was later discovered that the baby had no suckling reflex. Similar experiences were also corroborated in discussions, where female caregivers often reported providers discounting their perspectives or concerns completely.

“*When you ask how the baby is, the nurses tell you don't you have eyes? Others say it's your child you can go check yourself, you are the one who gave birth to him, you are the one who knows, us we don't.”* FGD Women with young infants (0–59 days)

“*The person taking the temperature annoyed me saying how my baby was tiny, how I don't feed the baby well …my baby had rickets. The doctor also accused me of not taking good care of my baby… I tried to explain the baby's history to the doctor, but he said, ‘wee mama enda useme hizo story uko mbele' (you mother, go and tell those stories elsewhere)”*. FGD Women with young children (60 days to 24 months)

Another negative experience was illustrated when older children were receiving care, the likelihood that they might inadvertently remove treatment “tubes” (such as intravenous lines (IV), nasogastric feeding tubes, or oxygen lines) were high, given the infants were uncomfortable. However, when mothers requested for a provider to assist in re-inserting the IV or raised a concern during care, they were ignored.

“*The baby was stubborn, inpatient, and kept removing the oxygen (nebulizer) because that thing irritates. He was also put on a drip since he had not eaten for days, which he also removed. There was another doctor who came when the baby had removed the drip, I informed him but no action of returning it was taken … I asked him “you cannot assist me to return his drip in place?” He told me in a tone that I did not like to carry my baby and move over there, where blood was spilling all over from his hand.”* FGD Women with young children (60 days to 24 months)

In addition to having their perspectives discounted, some women described being neglected. From the observations data, a baby was admitted to the pediatric ward at 11.45 a.m. and was left in the admission room for hours without being attended to. It took the intervention of the study team for the child to be attended to by a doctor at 3.00 p.m. Similar experiences were also documented in focus group discussions when women reported laxity amongst providers where requests for emergency care for their child were ignored.

“*There is also another time I came, and I had an issue, the baby was vomiting and having diarrhea. They just watched him vomiting and did nothing, after seeing the doctor, I expected him to give him medicine to stop the vomiting before providing other services, but they just wrote for me the drug. I went to the pharmacy, but the drug was not there. So, it forced me to go and look for it outside.”* FGD Women with young children (60 days to 24 months)

On some occasions, it was perceived that female caregivers with infants in newborn units received better care compared to those in general postnatal wards, and those that underwent surgery of any form were more closely monitored than those that did not. Other subtle forms of discrimination included failure to attend to women who gave birth at home or in another health facility.

“*You find that someone has been referred from another hospital to come there, you see the nurse quarrelling with her-why didn't you come to deliver here, you went to deliver elsewhere and when you get problems is when you are coming here. So, they consider the people who gave birth there first.”* IDI Couple with a young child (60 days to 24 months)

Lack of privacy during various physical examinations was also observed, although there were curtains and screens available, they were rarely used while attending to sick children, instead providers would examine the child in the open area where everyone could hear what was being discussed. During observations in the pediatric ward, a mother came with a child one and a half years old who had 9% burns on her back. She was allocated a bed and the nurse told her to wait for a while as she went to prepare for dressing. Since the baby was naked, the other caregivers freely looked at the child's burns on the back, even older children playing around came to see, but there was no intervention from the nurse. The nurse slowly prepared for the dressing for about 30 min, meanwhile the child was naked, and it was a cold afternoon. The child cried in excruciating pain as the nurse peeled off the burnt skin on her back. She did not calm or comfort the child in any way during the process or explain the procedure to the mother. This happened openly where any caregiver and other children could see.

We single out communication as part of interaction with providers to describe their experiences of care along the journey of hospitalization. Female caregivers reported minimal communication with providers during inpatient care. For newborns, providers shared information on weight gain but rarely explained how the newborns were progressing or responding to treatment. For young children aged 60 days to 24 months, caregivers mentioned having limited understanding of their child's illness, causes, or danger signs; they also felt providers lacked patience and time to communicate adequately due to their heavy workloads. Often medication was prescribed with limited explanation of test results or diagnosis.

“*What l feel is wrong is … if you want to know how your baby is fairing on because even your husband can call and ask how the baby is doing and you don't know, or you have been answered in a way that made you want to cry and you only wanted to know how your baby is doing*.” FGD Women with young infants (0–59 days)

“*They did not explain anything to us, they just told us if I can agree they give the baby three injections…but now they had tested and found no infection, how come they wanted to give the baby three injections?”* IDI, Couple with a young child (60 days to 24 months)

#### Perspectives from male caregivers

We did find that male caregivers had slightly different experiences to female caregivers. Men across both counties experienced barriers to being involved in caring for their sick young children. Due to the facility constraints and infection control protocols in public hospitals, men were not allowed into the newborn units and were often rebuffed by providers who were too busy to engage with them. On some occasions, providers did not appear to perceive men as active decision makers in the care of their sick child.

“*When I came to visit my wife and baby, the doctors came and found us together, … they came to call my wife that the baby was crying, I asked if I could see him (the baby), but I was not allowed, they (doctors) just told me ‘I have only called your wife and I need her to come and attend to what I have called her for' … I have never spoken to a doctor*.” FGD, Men

“*For me, I just hear that they are rude, you can't even ask a question about your child, you will be answered in a rude way”* FGD Men

Discrimination based on appearance, level of education, or ability to pay was also reported by men as an issue while seeking care. Men in one rural setting reported that if they presented to the facility looking unkempt, they would be treated poorly while in urban settings, men reported that those who could pay or were perceived to be influential were treated better.

“S*ome of the doctors check how the parents have come in, the condition, your dressing- when a parent comes with a child, he (the doctor) looks at the cleanliness of that child, … and he might see you as poor, and treat you according to how you have come*” FGD, Men

“*The ambulance is supposed to serve everyone. There is a time my baby was taken to [the hospital] for some test then brought back. In the middle of the journey, the driver stops the ambulance and demands for money. He even asks you to step down from the ambulance, yet you have signed and authorized documents. I felt so bad. That should not happen*.” FGD Men

Similar to female caregivers, when it came to communication with providers, male caregivers also frequently complained that providers did not share the condition of the child, they were rarely given information on how their child was responding to medication and results of tests during inpatient care.

“*When she was taken to nursery we could have been called together and be explained to. And we decide … They are supposed to explain to us what they are doing. Sometimes they were attending to the baby, and you do not know what they were doing to the baby, so they should explain*.” IDI, Couple with a young infant (0–59 days)

“Y*ou would like to know how the baby is doing. The frustration of having your wife there, the baby's condition also varies every day, like our baby's condition now at some point he dropped from 1.9 to 1.6 kgs, I could not ask my wife about such questions as she will not know anything because she is very confused about the whole situation, and she might not want all those questions all the time*.” IDI, Couple with a young infant (0–59 days)

### Emotional support

Emotional support was more pronounced during inpatient care rather than admission, outpatient care or discharge. To help cope with hospitalization, the study team observed caregivers forming new friendships with other caregivers who had already spent several days in the hospital. Experienced caregivers welcomed new caregivers, shared their experiences at the hospital, advised on how to interact with staff, and offered to watch their neighbor's child while caregivers went to bathe or take care of other issues. In addition to peer support, in the faith-based facility, providers sought out the chaplain to offer spiritual support to caregivers in the event of a child's death.

In one case we observed where a mother whose 1 year and 3 months old child was admitted due to severe malnutrition. The mother did not have adequate support from her husband or family members. Her husband had come once since admission and never returned. Nurses had instructed her relatives not to come because they would always discourage her and suggest that the child should be discharged and let to die at home, which left the mother feeling more distressed. Facility management suggested that that the mother would be linked with a social worker to assess her financial situation and help her get any resources she may need during her time at the hospital. The mother was elated on hearing the assurance that they would assist her with diapers and help her visit an ante-natal clinic because she was 8 months pregnant and had only attended clinic once.

Emotional support in general was not common in public facilities; on many occasions, the research team observed minimal, or no support given to caregivers who had experienced a difficult diagnosis or loss. For example, one infant who died was taken to a sluice room, wrapped in a bed sheet, and placed on a slab with no explanation given to the caregivers or no acknowledgment from the provider on the loss of life. Both male and female caregivers reported the emotional toll of hospitalization.

“*My baby was born here, so while at the nursery can you imagine the doctor came and told my wife my baby was born with brain damage … so my wife called me, I had to come and talk to her cause she was in shock, she was just crying*” FGD, Men

“*There is a time a mother lost her baby at the emergency room. I did not see anyone talk to her; she was by herself. She was trying to call her brothers. And she stayed there alone till morning no one bothered. But the baby had been taken to mortuary. I didn't see any counseling done to her*.” FGD Women with young children (60 days to 24 months)

## Discussion

This study used qualitative methods comprising interviews and ethnographic observations to explore and triangulate caregivers' experiences of care related to their sick young children while in hospital settings in Kenya. It is one of the few studies to focus on caregivers' experiences in sub-Saharan Africa, and to unpack mistreatment from an intermediary parent perspective (between service providers and sick young children), though we did not seek to classify mistreatment. The findings highlight how inadequate hospital infrastructure and complex hospital systems can frustrate caregivers along the journey of care from admissions to discharge. Negative experiences around interaction with providers, limited communication, and lack of emotional support all hinder the delivery of quality pediatric care. We also found similarities and differences between male and female caregivers that warrant further attention when designing and implementing interventions to improve experiences of care.

### Challenges of navigating the hospital environment impedes positive experience of care

We identified challenges around navigating hospitals' structural environments and payment systems as a major cause of negative experiences for both female and male caregivers. Poor signage, prolonged waiting times, staffing shortages, complex processes of care, hospital policies, and lack of communication on hospital environment and policies all resulted in poor experiences of care. These findings are consistent with a study in Malawi that found long waiting times and late facility opening times were some of the critical health system factors affecting healthcare seeking for under-5 children (24). The WHO standard for improving quality of pediatric care states “that the flow of patients must not be a barrier to accessing urgent care, and administrative or payment procedures must be delayed until the child has been started on medical care” (10). In this study we found caregivers visiting 5–10 service points while seeking emergency care in both inpatient and outpatient departments (see Figure 1 for the client flow journey). Delays at each of these service points affected access to prompt urgent care for sick young children and made male caregivers feel like they had to bribe providers to navigate the system. Another study in Malawi found delays between seeing different health workers once at the facility led to perceptions of low quality of care among clients (25). In Ghana, unclear hospital practices led to caregivers using negotiating strategies including “getting to know someone”, “exchange of favors” and “being appreciative” to access care (26). While in Nigeria, health workers mentioned that under the table payments were quite prevalent in secondary and primary care level facilities (27). Some ways stakeholders suggested combatting the issue was to have bottom-up approaches such as increasing patient involvement and strengthening the voice of services users and top-down approaches like more transparency on costing of drugs and services. Enhancing hospital navigability and increasing awareness of hospital policies and processes may improve caregiver experiences. One study in Canada described how a multi-disciplinary quality improvement team involving frontline staff and clients improved facility navigability by using floor graphics/arrows and welcome signs outlining registration procedures (28). The study in Canada highlights potential opportunities to implement low-cost, structural changes that may improve parent satisfaction when seeking care for sick young children in large hospitals.

In this study we identified how limited infrastructure has also exposed female caregivers to overcrowded facilities and possible infections, hampering their ability to meaningfully engage and care for their child. The implications of these findings highlight the need to address structural issues and develop holistic sustainable solutions to ensure caregivers and their families remain central to care. In both Bungoma and Nairobi, shortages in nursing workforce, limited infrastructure, poor leadership, governance, and other issues have undermined the quality of care provided for sick young children (20, 21, 29, 30). Ensuring providers are working in an enabling hospital environment that addresses staffing shortages, provides a supportive environment that includes mentorship, teamwork and supportive supervision as well as ensuring there are adequate supplies and equipment for providers to carry out their duties can improve the quality of care.

### Female and male caregiver interactions with providers affects understanding of treatment processes and partnerships in care

There are clear links between gender and health care seeking for sick young children (31). In traditional African societies caregiving is seen as a woman's job tasked with feeding and nurturing the child while the man is tasked with providing shelter, food and controlling resources when deciding to seek healthcare. These disparities are also seen in hospitals where for young children, women are viewed as the primary caregivers for sick young children however they most often do not make independent decisions on care and usually must consult men or other individuals in their social circles. Considering the changing dynamics of child-care, evidence around importance of male involvement in reproductive, maternal, newborn and child health (32) fathers are encouraged to play an active role in caring for their children, and we see male involvement in skin-to-skin care seen as both feasible and beneficial to infants and fathers (33). Though much of the gender literature in this space focuses on decisions to seek care, less has been done to understand similarities and differences in experience of care between female and male caregivers while receiving care for their children in hospital settings.

In this study we have demonstrated that poor caregiver-provider interactions resulted in caregivers not being treated with respect and dignity. Female and male perspectives were similar in terms of experiencing harsh language from providers, or providers discounting caregiver perspectives when they expressed concern about their child's care. Verbal abuse of women during childbirth and caregivers being blamed for poor neonatal outcomes is increasingly described globally and in Kenya, including pathways of influence (14, 16, 17, 34). This study identifies another aspect of mistreatment of caregivers of children 60 days to 24 months previously unreported, namely, that caregivers are ignored when they ask for medical help such as re-inserting treatment tubes that are pulled out by children. Previous research has shown that the impact of negative previous experiences and provider attitudes can result in caregivers losing trust in health services and can negatively influence future care seeking behavior for under-5 children (8, 24, 27).

Some differences between female and male perspectives of interactions with providers include the lack of attention to the mother-newborn dyad and poor provider engagement of men in sick young children's care. Female caregivers reported providers only focused on emergency care for the woman and ignored the newborn or in some cases ignored both woman and her newborn. These findings go against the “Every Newborn Action Plan” that calls for interventions targeting both women and newborns to reduce preventable deaths (2). We documented how men were often side-lined and did not feel comfortable asking questions about their child, resulting in them feeling isolated and out of the loop in understanding their child's illness. Providers' apparent reticence to involve men in the care of their children was especially notable in the public health facilities and consistent with findings in Uganda and Ghana (35, 36). Similarly, fathers in these studies although were willing to participate in care felt excluded resulting in increased stress and lacking confidence to care for their children once back at home. Studies in East Africa have shown that providers who are not adequately trained to provide male-friendly services and stereotyping and feminization of reproductive and MNCH issues are key barriers to effective male involvement (32, 37). Another key difference in female and male perspectives was discrimination based on socio-economic status. While female caregivers reported discrimination because of birth location, men noticed that appearances and a lack of money determined the kind of care they received. These findings are consistent with other studies in Kenya that identified providers having implicit and explicit bias based on wealth/social status leading to differential treatment during childbirth (13, 14, 38).

In addition to poor interaction between providers and caregivers, limited communication by providers was reported by both female and male caregivers and hindered their ability to meaningfully participate in care. This is in line with a study of seven countries in Sub-Saharan Africa that found that only about half of caregivers reported they were informed of their child's diagnosis and only 10% reported they were counseled on child feeding (39). With sick young children (0–24 months) unable to verbalize their experiences of care, caregivers play a significant role in determining both the presence and severity of symptoms. Communication with providers, the avenue through which this can be achieved, warrants targeted intervention and support for both providers and caregivers. WHO guidelines call for children and their caregivers to be given understandable information during care and follow-up about the disease or condition, potential long-term effects, and how to access further support (10).

### Limited emotional support impedes positive coping mechanisms for caregivers with hospitalized children

Throughout their hospitalization journey, inadequacies of emotional support affected caregivers' ability to cope with stress and anxiety related to hospitalization and their understanding of the treatment process and outcomes. Lack of bereavement and counseling was identified by both male and female caregivers as a cause of negative experiences and is similar to findings in Ghana (26). Informal support from their peers was important in helping to deal with negative experiences of care. Although emotional support from providers were limited, they were instrumental in how caregivers coped with hospitalizations. This is consistent with research on stillbirths, which found providers who displayed positive behaviors and actions, such as non-verbal expressions of touch, listening, and letting caregivers express themselves, may have memorable impacts (40). In addition, emotional and peer support groups for caregivers were found to be an important facilitator for children with disabilities and their caregivers accessing healthcare services in Sub-Saharan Africa (41). In this study we found two dimensions of emotional support being practiced (1) peer to peer support that helped with understanding hospital processes and (2) linking caregivers with chaplains, social workers, or counselors within the health facility. These avenues also need to inform interventions on nurturing care that have already been adopted by the Government of Kenya to improve early childhood development.

These findings while similar in other low- and middle-income countries they highlight the need to look beyond quality of care and focus on interventions that will improve experiences of care during the journey of hospitalizations. We presented these findings to healthcare providers who work in these facilities, caregivers who sought services in these facilities as well policy makers and other stakeholders, and many of the recommendations presented here were suggested by these stakeholders during a dissemination and co-creation workshop to design a structural and behavioral change intervention to provide nurturing care to hospitalized newborn and young children 0–24 months.

Interventions required to overcome barriers to quality of care identified in this study, including provider attitudes, structural policies restricting male involvement, poor communication and rapport between providers and caregivers, and inadequate emotional support. This study shows that eliciting both female and male perspectives to improve quality and respectful care of newborns/young children is not only important but may need to be considered in intervention and program strategies. The WHO stipulates that to help children in the early years of their life reach their full potential, they need five inter-related and indivisible components of nurturing care: good health, adequate nutrition, safety and security, responsive caregiving, and opportunities for learning (42). The Kenyan Government as mentioned above has adopted nurturing care as part of early childhood development. The Newborn, Child, and Adolescent Health policy calls for nurturing care interventions to be included in child health programming, however, these have not been fully financed or operationalized (43). Other partners have also documented that critical nursing workforce shortages will continue to prevent delivery of high-quality newborn care and likely threaten patient safety and nurses' wellbeing (20). Future intervention activities to address negative experiences of caregivers, such as training and motivating health providers on nurturing care, coaching caregivers on responsive care (where they notice, understand, and respond to their child's signals in a timely and appropriate manner), targeting men, improving communication with caregivers, and emotional and peer support for providers and caregivers may be helpful in empowering female and male caregivers to better communicate with providers in a hospital setting. They may also have a secondary effect on enhancing their trust and confidence in health services more broadly. The evidence from this study informed an intervention design for a pilot intervention strategy inclusive of the above, that seeks to improve experience of care for caregivers and their sick young children (0–24 months).

## Strengths and limitations

A strength of this study is the use of multiple methods (IDIs, FGDs, and ethnographic observations) to elicit and triangulate issues of experiences of care specific to female and male caregivers. Although men were difficult to recruit for FGDs, they were more likely to agree to participate in a one-to-one interview, therefore they were included in both the joint IDIs and FGDs. A limitation of the study was we only collected data in five large hospitals which may not necessarily reflect experiences in all hospitals in Kenya. However, these experiences may be applicable to secondary and tertiary institutions, which have similar characteristics. This study focused on experiences of care for caregivers of sick young children in hospitals. Therefore, other types of mistreatments, such as from providers at primary health care facilities, at community level from community-based health care workers, or lack of support within families are not included.

## Conclusion

This study describes the experiences of care that were observed and/or reported for caregivers of sick young children in five hospitals in Kenya. Positive and negative experiences were identified and affected how caregivers navigated the hospital systems, interacted with providers, and supported and cared for their sick child. This is one of a few studies in Kenya that looked at caregiver perspectives of caring for one's hospitalized child, including how exposure to similar and ass different experiences of women and men can overlap to undermine the trust and confidence in health services. There needs to be concerted effort to meaningfully involve both female and male caregivers in the care of their children while in hospital. Improving communication between providers and caregivers, emotional support for caregivers, and providing supportive, enabling, and healthy environments will lead to more respectful care of sick young children and their caregivers.

## Data availability statement

The data that support the findings of this study are available upon request from the corresponding author CO. The data are not publicly available, as they contain information that could identify the research participants.

## Ethics statement

This study was approved by the Population Council's Institutional Review Board (PC IRB 893) and the African Medical and Research Foundation (AMREF) Ethics and Scientific Review Committee (P646/2019). Researchers were trained to ensure that guidance on ethical conduct was clearly understood and implemented. Written informed consent was translated to Kiswahili, read to potential participants, and once understood and accepted, participants signed the informed consent form before participating in the study. Interviews with caregivers were conducted in English or Kiswahili depending on the fluency of the participants, whilst all FGDs were conducted in Kiswahili. The research team was trained to listen and observe intently without displaying any judgmental attitude. In cases where caregivers reported undue stress during data collection, they were referred to psychological support. One mother in Bungoma and another in Nairobi were referred for further psychological support within the health facility structures.

## Author contributions

CW: conceptualization. CW, TA, CN, and PS: designed the experiments. CN, CO, and TA: conducted the study and analyzed the data. CW and PS: contributed analysis tools and conceptualized and guided development of the manuscript. CO, TA, CW, PS, and CN: contributed to the interpretation of the results and writing—review and editing. CO: original draft of paper. All authors contributed to the article and approved the submitted version.

## Funding

This study was funded by the generous support of the American people through the United States Agency for International Development (USAID) under Breakthrough RESEARCH (Cooperative Agreement No. AID-OAA-A-17-00018).

## Conflict of interest

Authors CO, CN, and TA were employed by Population Council, Nairobi, Kenya. Authors PS and CW were employed by Population Council, Washington, DC, United States.

## Publisher's note

All claims expressed in this article are solely those of the authors and do not necessarily represent those of their affiliated organizations, or those of the publisher, the editors and the reviewers. Any product that may be evaluated in this article, or claim that may be made by its manufacturer, is not guaranteed or endorsed by the publisher.

## Author disclaimer

The contents of this manuscript are the sole responsibility of the authors and do not necessarily reflect the views of USAID or the United States Government or the Kenyan Government.

## Footnotes

^1^This was developed from the observations data and further refined in a co-creation workshop where parents and providers shared their perspectives navigating the hospital system.

## Abbreviations

FGD: Focus group discussions
IDI: In-depth interviews
MNCH: Maternal, newborn and child health
WHO: World Health Organization.
