# Supplementary material for: “You can't even ask a question about your child”: Examining experiences of parents or caregivers during hospitalization of their sick young children in Kenya: A qualitative study
Source: Front Health Serv. 2022 Oct 3;2:947334. doi: 10.3389/frhs.2022.947334 (PMC10012665; doi:10.3389/frhs.2022.947334)
Supplement: Supplementary file 1 [file Table_1.DOCX]

CHILD HEALTH

Nodes

| Name | Description |
| --- | --- |
| Caregiver involvement in facilities | How caregivers are involved in care of children at facility level |
| Barriers to caregiver involvement | States limitations of involving caregivers in care of SYC |
| Caregivers’ involvement in care of SYC | This node states ways in which they are involved |
| Suggestions for more involvement of caregivers in care | Describes ways in which caregivers can be further involved in care of children at facility level |
| Experience of care | Describes available services and caregiver perceptions of the care received |
| Provider-caregiver interaction | How providers interact with caregivers during care seeking for SYC |
| Availability of Question-and-Answer sessions | Whether caregivers are allowed to ask questions and if they are answered to their satisfaction |
| Feedback mechanism and conflict resolution | How or whether conflict between caregiver and provider is resolved |
| Negative interaction | Description of negative interaction |
| Positive interaction | Illustrations on positive interaction |
| Provider-Child interaction | How provider treat or interact with SYC during provision of health care |
| Negative interaction | Illustrations of negative interactions between providers and children |
| Positive interaction | Illustrations of positive interactions between providers and children |
| Quality of services | Perceived value/worth of services provided |
| Caregiver and community perception | The caregivers/community’s view of quality of care offered at different facilities |
| Private and faith-based facilities | Quality of care offered in private and faith-based facilities |
| Public facilities | Quality of care offered in public facilities |
| Preferred time of services Day /Night | Caregivers preferred time and day to receive quality services |
| Priority given to emergency cases | Whether emergency cases with SYC are prioritised when care seeking |
| Process of care | Perceived processes of quality of care |
| Provider perception | The providers view of quality of care offered at different facilities |
| Time taken to be served | How long it takes to receive desired services |
| Referral | Refers to the general processes of referral |
| Challenges during referral | Illustrates challenges encountered during referral |
| Delays in initiating care by providers | How delays in initiating care affect the referral process |
| Discrimination of babies | How babies are discriminated during referrals |
| Distance | States influence of travelling long distances on the referral process |
| Financial constraints | This node states impact of lack of finances on the referral process |
| Inadequately equipped ambulances | How ambulances that are not adequately equipped affect the referral process and outcomes |
| Lack of transportation | How the availability of a means of transportation affects the referral process e.g. lack of an ambulance |
| Terrain | States influence of poor terrain on the referral process |
| Outcome of referral | Refers to the result of referral |
| Views on referral process | Perceptions on the referral processes |
| Referral from lower-level facilities | Describes the views of caregivers while their sick children are referred from other facilities to the site facilities |
| Referral to higher level facilities | Describes the views of caregivers while their sick children are referred from the site facilities to higher level facilities |
| Services at community level | Services offered at community level |
| Community perception on the services provided by facilities | Perception of community members think or feel about the services provided in facilities |
| Home visits | Illustrates health workers visiting caregivers of SYC in their homes |
| Outreaches | Meetings organized by facilities to provide health services to the community |
| Who offers services | Categories of people who provide these services |
| Services offered at facility level | Types of services offered at facility level |
| ANC | Describes services available for pregnant women |
| Delivery | Describes delivery services offered at facility level |
| Growth monitoring | Describes types of child welfare clinic services offered to children |
| Health education for caregivers | Health information shared with caregivers by providers |
| Immunization | Types of immunization services offered |
| KMC | Services offered for preterm babies and caregivers to enhance growth |
| Lab services | Types of lab services offered in health facilities |
| NBU_NICU_Inpatient services | This describes services offered to sick babies during hospitalization |
| Nutritional Services | Facilities provide nutritional education, foods and supplements |
| Outpatient services | Treatment and services offered to sick children on outpatient basis |
| Special clinics and follow ups | Scheduled follow up clinics for children born with complications but stabilised and discharged from facilities |
| Support for care of newborns and SYC | Type of support need or available for caregivers of SYC |
| Counselling | Suggestions to provide counselling services to caregivers with sick children |
| Financial Support | Provision of support to caregivers of sick young children through bill waivers |
| Linkage for continuum of care | Ways of improving linkages for continuum of care through satellite clinics or CHVs |
| Planning for child follow up | Improvised ways to improve follow-up for sick young children |
| Social and spiritual support | Respondent describes social assistance needed during receiving of health care, i.e., taking care of other children at home, visiting during hospitalization etc. |
| Support groups | Initiation of support groups for caregivers with children with similar conditions for the purpose of interaction and support |
| Family involvement in care for newborns and SYC | Ways in which general family members can/ are be involved in care for newborns and SYC |
| Decision making | How families to sick young children are involved in decision making during procedures and treatment |
| Emotional support | How family can emotionally support caregivers of SYC |
| Financial support | Describes how families stand in provision of finances to those with SYC |
| Social support | Ways in which family can offer social support to caregivers of SYC |
| Mistreatment_Poor Quality Care | This code describes how caregivers or children are treated in an inhumane way while seeking care |
| Caregivers coping mechanism for mistreatment | Ways in which caregivers deal with mistreatment |
| Drivers of mistreatment | Influences of mistreatment |
| Caregivers | This code describes ways in which caregivers of children participate in mistreatment of babies or children |
| Cultural influence | Influences of culture or cultural barriers that facilitate mistreatment |
| Delays in care seeking | Parents take long before deciding to seek care for newborns and sick young children |
| Fear of providers | Apprehension amongst caregivers to interact with providers e.g., to ask questions |
| Lack of avenue to channel complaints | Lack of appropriate means to express dissatisfaction with care e.g., Suggestion box |
| Lack of finances | Providers lack money needed to carry out treatment or procedures |
| Lack of knowledge | Caregivers’ illiteracy or lack of awareness on proper childcare |
| Negligence |  |
| Mistreatment by age category (0 to 28 day; 29days to 12 months; 13months to 24 months) | Perceived age to more likely be mistreated |
| Mistreatment by time of care seeking (weekends; night shift) | How different times of seeking care affect the quality of care given or mistreatment experienced. |
| Effects of mistreatment | How poor quality of care/ mistreatment affect caregivers of SYC or newborns |
| Adherence to treatment | How mistreatment affect caregiver’s response to adherence to treatment |
| Care seeking practices | Influences of mistreatment in alternative source of care and timely care seeking practices |
| Caregiver initiated care | Care initiated by caregivers |
| Further complications and Infections amongst infants and caregivers | How mistreatment results in further complications and infections for infants and caregivers |
| Infant mortality | How’s mistreatment results to infant or child deaths |
| Longer hospitalization | How mistreatment leads to longer hospitalization |
| Psychological emotional | Extent in which mistreatment affects caregivers emotionally |
| Relationship with providers | Effects of mistreatment on the relationship between providers and caregivers |
| Manifestation of mistreatment of babies | How mistreatment is demonstrated or expressed by babies |
| Abandonment by caregivers | Babies being rejected by caregivers |
| Delayed discharge | Babies are delayed when it comes to their discharge |
| Delayed services | Delayed initiation of care and how it is viewed as a mistreatment |
| Discrimination of babies | Illustrates instances where babies are treated with some form of biasness |
| Drug shortage | Lack of drugs needed for care and treatment of babies |
| Frequent pricking | Describes how newborns and sick young children are pricked severally which may be by unqualified health providers |
| Lack of adherence to standard of care | Illustrates providers inadequacy in observance of laid down procedures of health care |
| Negligence | Describes; laxity, abandonment, or disregard of babies during seeking of care at facilities |
| Overcrowding | Babies are crowded in cots or incubators |
| Physical abuse | Describes where unnecessary physical pain is inflicted on a baby’s body |
| Poor feeding practices | Babies not feeding on time or completely |
| Rough handling of babies (not gentle) | Describes where babies are handled roughly during care |
| Wrong diagnosis and medication | Participants describe the causes and influences of misdiagnosis |
| Manifestation of mistreatment of caregivers | How mistreatment is demonstrated or expressed by caregivers |
| Caregiver blaming by provider | Illustrates how caregivers are blamed for babies’ conditions by providers |
| Delayed services | Delayed initiation of care and how it is viewed as a mistreatment |
| Discrimination of care seekers | Perceived prejudice/ unfair treatment by providers |
| Ineffective /inadequate communication | Illustrates inadequate sharing of information between providers and caregivers |
| Lack of adherence to standard of care | Illustrates providers inadequacy in observance of laid down procedures of health care |
| Denial of caregiver access | Node describes instances where caregivers are denied access to SYC or partners |
| Withholding of services care | This describes service or care withheld during care seeking at facility level |
| Lack of emotional support | Refers to instances when emotional support is not offered to caregivers |
| Long queues | Illustrates long queues is viewed as mistreatment |
| Negligence | Describes; laxity, abandonment, or disregard of caregivers during seeking of care at facilities |
| Perceived corruption or request for bribes | Where caregivers were directly or indirectly asked to give a bribe to obtain services |
| Poor or crowded facility conditions | Describes congestion and poor facility conditions |
| Unresponsiveness to caregiver concerns | Unresponsiveness to caregiver concerns |
| Use of harsh or language tone | Providers use of inappropriate tone or language while addressing caregivers |
| Recommendations | Suggestions on improving care for newborn and sick young children |
| Availability of round the clock services | Availing of services day and night |
| Conducting more outreaches | Outreaches conducted by facility staff to ensure children below 2 years are in good health |
| Improved provider caregiver relationship | Ways in which provider and caregivers’ interactions can be improved |
| Provision of adequate resources at facility level | Support by the government to ensure adequate supply of resources (drugs, essential supplies and human resource |
| Responsiveness_ Fast response | Refers to timely feedback and action |
| Roles in child health care | Defined roles played by caregivers and other stakeholders in child health care |
| Role of administrators and health managers | Role of administrators and health managers in the facilities |
| Role of CHEWS_CHVs | Role of the CHEWs and CHVs in ensuring dignified or proper care in SYC |
| Role of community members | Role of other community members in child health care |
| Role of county and national governments | Responsibility of the county and national government in child health care |
| Role of developmental partners | Role of partner organisations in child health care |
| Role of family | The responsibilities of family members in child health care |
| Role of health care providers | Role of health care providers |
| Role of mothers | Responsibility of mothers and female caregivers in child health care |
| Roles of fathers | Responsibility of male parents and caregivers in child health care |
