# Supplementary material for: “You can't even ask a question about your child”: Examining experiences of parents or caregivers during hospitalization of their sick young children in Kenya: A qualitative study
Source: Front Health Serv. 2022 Oct 3;2:947334. doi: 10.3389/frhs.2022.947334 (PMC10012665; doi:10.3389/frhs.2022.947334)
Supplement: Supplementary file 2 [file Table_2.DOCX]

**FGD guide for Parents of Sick Children**

| **PARTICIPANT NUMBER** | **PARTICIPANT’S SEX**  **(MALE OR FEMALE)** | **PARTICIPANT’S AGE**  **(YEARS)** | **CHILD’S AGE** |
| --- | --- | --- | --- |
| Participant 1 |  |  |  |
| Participant 2 |  |  |  |
| Participant 3 |  |  |  |
| Participant 4 |  |  |  |
| Participant 5 |  |  |  |
| Participant 6 |  |  |  |
| Participant 7 |  |  |  |
| Participant 8 |  |  |  |

**Note:** *Small and sick newborns for this review refers to a newborn who is born preterm, small for gestational age, has an illness, or suffers from a birth complication, and requires hospitalization during the neonatal period.* ***Young children for this discussion refers to children anywhere from birth to 2 years old.***

*Watoto wadogo na watoto wachanga wagonjwa hapa inamaanisha mtoto mchanga aliyezaliwa kabla ya watati wake kufika, mdogo kwa umri wa ishara, ako na ugonjwa ama ana shida tangu kuzaliwa na anahitaji kulazwa hospitalini punde tu baada ya kuzaliwa.* ***Watoto wadogo hapa inahusu watoto kati ya umri wa kuzaliwa hadi miaka miwili.***

1. Let us talk about the services you receive at this facility for newborns and young children 0-2 years of age? **Probe for** childbirth, newborn, postnatal care and young children services. Are their services you would like to receive for young children but have not received?

*Sasa ningependa tuzungumzie huduma ambazo unapokea kwa hiki kituo cha afya kwa ajili ya watoto wachanga na watoto wadogo kati ya miaka 0-2.****ulizia*** *kujifungua, watoto wachanga, huduma za baada ya kujifungua na huduma za watoto wadogo. Je kuna huduma zozote za watoto wadogo ambazo ungependa kupokea lakini haujapokea?*

1. Tell us about a typical scenario from the time you seek services for a baby/child 0-2 years till the time you leave the facility.

*Tueleze kuhusu hali ya kawaida kutoka ule wakati unatafuta huduma kwa ajili ya mtoto miaka 0-2 hadi ule wakati unatoka kwa kituo cha afya.*

1. What is your perception about services provided for newborn and young children 0-2 years in this community, sub-county, county and national government? **Probe for** routine services and sick children of all ages 0-2 years.

*Je unafikiriaje kuhusu huduma zinazopeanwa za watoto wachanga na watoto wadogo miaka 0-2 kwa hii jamii, kata ndogo ya kaunti, kaunti na serekali kuu ya kitaifa?* ***Ulizia*** *huduma za kawaida na watoto wagonjwa wa miaka 0-2.*

**Topic I: Understanding experiences of interactions between parents and health care providers**

***Kuelewa uzoefu wa mwingiliano kati ya wazazi na watoa huduma za afya***

1. Thinking back to your experiences when your baby was unwell, tell us what normally happens between parents and providers at health facilities – what are their interactions like?

*Ukifikiria kuhusu matukio ya wakati mtoto wako alikupowa mgonjwa, tafadhali tueleze ni nini hufanyika kwa kawaida kati ya wazazi na wahudumu wa afya kwenye vituo vya afya - je maingiliano yao yako vipi?*

**Probe for;**

**Ulizia:**

- ***What departments do parents visit?***

*Wazazi hutembelea idara/vitengo gani?*

- ***Are parents greeted nicely? Do providers introduce themselves?***

*Je wazazi husalimiwa vizuri? Je watoa huduma hujitambulisha?*

- ***Do providers explain how to care for one’s child? Do they explain what needs to be done (e.g. tests) in a timely way in which is understandable?***

*Je watoa huduma hueleza jinsi ya utunzaji wa watoto? Ni mambo gani ya kuangalia km vipimo kwa wakati uliosawa kwa njia ambayo ungeelewa?*

- ***Are parents allowed and able to ask questions? Are their questions answered satisfactorily?***

*Je wazazi wanaruhusiwa kuuliza maswali? Je maswali yao yanajibiwa na wanaridhika?*

- ***What types of instructions about follow up care do providers give after a baby’s discharge?***

*Ni aina gani ya maagizo kuhusu utunzaji wa kufuatilia ambao wahudumu wanapeana baada ya mtoto kutolewa hospitalini?*

- ***How did the providers speak to you - in a friendly tone? Rushed tone?***

*Je wahudumu walikuongelesha vipi – kw asauti ya kirafiki? Kwa sauti ya hasira?*

1. In your opinion, have seen or heard of any disagreements or challenging moments with the service providers during your child’s care. **Probe:** Please describe what happened and where? How are these instances resolved? How do they affect parents and their children?

*Kwa maoni yako, je umeona ama kusikia kutokukubaliana kokote ama matukio ya changamoto na wanaotoa huduma wakati wa utunzaji wa mtoto wako.* ***ulizia:*** *kama ndio tafadhali eleza ni nini ilitokea na wapi? Yalisuluhishwa vipi?je yanaathiri vipi wazazi na watoto wao*

- 1. ***Do you think parents like yourselves felt - at any point in your interactions with providers - humiliated in anyway while your sick child received care in this facility? Probe: what, how and Why***

*Je unafikiria wazazi kama wewe- wakati wowote wa maingiliano yenu na mhudumu wa afya- walihisi kudalilishwa(aibu) kwa njia yoyote wakati mtoto wako mgonjwa alipopokea utunzaji kwa hiki kituo* ***ulizia*** *nini, kivipi na kwanini?*

1. What type of help do parents need to effectively care for their sick child while in the hospital?

**Probe for - emotional support, instructions about childcare, information / counselling?**

*Ni msaada wa aina gani ambao wazazi wanahitaji ili watunze watoto wao wagonjwa kwa ufanisi wakiwa hospitalini?* ***ulizia:-*** ***msaada wa kihisia, maagizo ya utunzaji ya mtoto,je maagizo au mashauri?***

1. ***Do parents receive any other support? If yes what type of support?***

*Je wazazi hupokea msaada wowote? Kama ndio msaada wa aina gani?*

1. ***Who supports parents and families during hospitalization?***

*Je ni nani ambaye hupatia wazazi na familia msaada wakati wamelazwa?*

1. ***What support do parents require in the long-term?***

*Wazazi wanahitaji msaada wa aina gani kwa muda mrefu ujao?*

1. **Are parents ready and or able to follow-up with the instructions/ counselling given by the provider?**

*Je wazazi wako tayari na wako na uwezo wa kufuatilia maagizo/ ushauri wanaopewa na wahudumu?*

1. What do parents need to assist them in short and long-term care of sick children 0-2 years? **Probe for: resources, who should be involved and how? (newborn 0-28 days and sick children 29days - 2 years)?**

*Wazazi wanahitaji nini ili kuwasaidia kwa utunzaji wa mda mfupi na mda mrefu wa watoto wagongwa kati ya miaka 0-2?****Ulizia;*** *rasilimali, ni nani anafaa kuhusishwa- kivipi na kwa nini?* ***(watoto wachanga siku 0-siku 28 na wagonjwa kati ya siku 29 – miaka miwili)***

**Topic II: Understanding caregiver context for respectful care for newborn and young children**

***Kuelewa mukthada wa walezi kwa ajili ya utunzaji wa watoto wachanga na watoto wadogo ulio na heshima***

1. Do you think providers try to build a relationship with parents of sick young children under the age of 2 years?

*Je unafikiria kwamba wahudumu wa afya wanajaribu kujenga mahusiano na wazazi wa watoto wadogo wagonjwa chini ya umri wa miaka miwili?*

- 1. ***How are newborns and sick young children are “treated” by the health providers i.e. do you think providers try to build a relationship with the babies?***

*Je, ni jinsi gani watoto wachanga na watoto wadogo wagonjwa wanahudumiwa na watoa huduma wa afya ie je unafikiria kuwa wahudumu wanajaribu kujenga mahusiano na watoto?*

- 1. ***Do you think providers treat babies as human beings? Please explain.***

*Je unafikiria wahudumu wanawahudumia watoto na utu (kibinadamu)? Tafadhali eleza*

- 1. ***Do you feel the provider is gentle with babies? Please explain.***

*Je unafikiria wahudumu ni wapole kwa hao watoto? Tafadhali eleza*

- 1. ***How do providers speak to parents of sick children?***

*Je wahudumu hao huwaongelesha vipi wazazi wa watoto wagonjwa?*

1. Think of all the different people who might have a role in making sure children 0-2 years are treated in a friendly, sensitive and humane way. What are the roles of: **parents/caregivers, fathers, families, community members, community health workers Community health volunteers (CHVs), healthcare providers, health managers and government county/national?**

*Fikiria kuhusu watu wote tofauti ambao wako na jukumu la kuhakikisha kwamba watoto wa miaka 0-2 wanahudumiwa kwa njia ya kirafiki, inayojali na njia ya utu/kibinadamu. Ni nini majukumu ya:* ***wazazi/ walezi, baba, familia, wana jamii, wahudumu wa afya wa kijiji, wahudumu wa afya wa jamii wa kujitoea, mameneja wa afya na serekali kaunti/ kitaifa?***

- 1. **How are caregivers prevented from caring for their babies? Probe: how is it different for men and women?**

*Ni jinsi ganzi walezi wanazuiliwa kutuko utunzaji wa watoto wao?* ***Ulizia:*** *unatofautiana vipi kwa wanaume na wanawake?*

1. Please can you give any example of insensitive or inhumane treatment for newborn and sick young children 0-2 years in a hospital? What does this poor treatment look like at: Maternity, OPD, Pediatric ward Post-natal ward? **Probe in, public, private; faith-based, referral hospitals**

*Tafadhali unaweza eleza mfano wowote wa huduma zisizojali au huduma ambazo si za kibinadamu(utu) kwa watoto wachanga na watoto wadogo wagonjwa kati ya miaka0-2 kwa hospitali? Je hii huduma mbaya inakaa aje kwa chumba cha wamama wenye mimba, OPD, wodi ya watoto na wodi ya baada ya kujifungua?* ***ulizia;*** *kwa hospitali za umma, hospitali za kibinafsi. Hospitali za imani, na/au hospitali za rufaa*

1. At what age are newborns and young children more likely to receive poor quality of care and/or insensitive/inhumane treatment?

*Ni katika umri gani watoto wachanga na watoto wadogo wako na uwezekano zaidi kupokea utunzaji duni na/ au huduma ambazo hazijali/ huduma ambazo si za kibinaamu(utu)?*

1. **Probe if differentiated by**: age group: 0-28 days; 29 days – 12 months and; 13 months – 24 months of life?

***Ulizia****: kama imetofautishwa na kikundi cha umri: siku 0-28, siku 29–miezi 12, miezi 13– 24 au maisha?*

1. What do you think might be reasons for poor quality of care, unfriendly, insensitive or inhumane treatment? Probe for: facility-related issues (e.g. space/infrastructure, staff, medicines/supplies, any other)

*Ni nini unafikiria inaweza kuwa sababu ya utunzaji duni usio wa kirafiki, usiojali au utunzaji usio na utu/wa kibinadamu?****ulizia****:mambo yanayohusiano na kituo cha afya (kwa mfano nafasi/ miundo mbinu, wafanyi kazi, madawa/ vifaa vya madawa, nyengine)*

- 1. **How does it work for routine outpatient care and inpatient services (e.g. NBU, NICU/pediatric and post-natal ward)?**

Inafanyaje kazi kwa utunzaji wa kawaidia kwa wagonjwa wasiolazwa na huduma za waliolazwa**( eg NBU, NICU/ wodi za watoto na wodi za baada ya kujifungua)?**

1. How does poor quality of care or insensitive / inhumane treatment of newborns and young children affect caregivers and families of newborn and young children 0-2 years?

***Probe for: Psychologically/emotionally, physically, health seeking behaviour, adherence to follow up instructions, relationship with providers, any other?***

*Je, ni jinsi gani utunzaji duni au utunzaji usio nyeti / usio wa kibinadamu (utu) wa watoto wachanga na watoto wadogo unaathiri walezi na familia zao?*

***Ulizia: kisaikologia/ ama kihisia, kimwili, tabia za kutafuta utunzaji wa afya,ikiwa au ikiwa sivyo wazazi hufuatilia maagizo,mahusiano kati ya wahudumu wa afya, nyengine?***

1. How could families be more involved in deciding what and how their newborn and sick young children (0-2 years) can be cared for? Probe for while receiving:

*Je, ni jinsi gani familia zinaweza kuhusishwa zaidi katika maamuzi ya ni nini na ni vipi watoto wao wachanga na watoto wadogo wagonjwa (0-2) wanaweza kupata utunzaji?*

***Probe for while receiving:***

***Ulizia wakati wanapokea:***

- 1. ***Maternity/postnatal ward***

*Wodi ya wamama wajawazito/ wodi ya baada ya kujifungua*

- 1. ***Inpatient services such as Newborn Unit (NBU), Newborn Intensive care unit (NICU), Pediatric ward***

*huduma za afya za kulazwa kama wodi ya watoto, kitengo cha watot wachanga, kitengo cha utunzaji mkubwa*

- 1. ***Ambulatory outpatient services for sick young children 0-2 years***

*Huduma za afya zinazopewa watoto wachanga ambao ni wagonjwa kati ya miaka 0-2 na hawalazwi hospitalini*

1. Do you think parents **should/can** do some of the things that nurses do (for example tube feeding, turning baby’s position, changing soiled linen, giving some medication, in newborn and pediatric service areas for children 0- 2 years – for example the neonatal unit/NBU? If yes **Probe** for:

*Je unafikiria kwamba wazazi wanafaa/ wanaweza kufanya mambo mengine ambayo wahudumu wa afya wanafanya (kwa mfano kuwalisha kupitia kwa bomba, kugeuza sehemu ya watoto, kugeuza pamba/ nguo zilizochafuka, kupeana madawa, kwa maeneo ya huduma ya watoto wachanga na watoto wadogo kati ya miaka 0-2- kwa mfano NBU? Kama ndio* ***ulizia:***

- 1. ***Which ones would you be happy to do yourself? Have you ever done them?***

*Ni zipi ungefurahia kufanya kibinafsi? Ushawai zifanya*

- 1. ***Would you be interested in being even more involved? Why or why not?***

*Je ungependelea kushughulishwa zaidi? Kwa nini au kwa nini sivyo?*

1. What strategies can we use to improve relationships between health providers and families of small sick babies and young children? **How can caregivers and providers work better together?**

*Ni mikakati ipi tunaweza tumia ili kuboresha mahusiano kati ya wahudumu wa afya na familia za watoto wadogo wagonjwa na watoto wadogo?****je, ni jinsi gani walezi na watoa huduma wanaweza fanya kazi bora pamoja?***

**Anything else to add?**

*Jambo lengine la kuongezea?*
