# Supplementary material for: “You can't even ask a question about your child”: Examining experiences of parents or caregivers during hospitalization of their sick young children in Kenya: A qualitative study
Source: Front Health Serv. 2022 Oct 3;2:947334. doi: 10.3389/frhs.2022.947334 (PMC10012665; doi:10.3389/frhs.2022.947334)
Supplement: Supplementary file 3 [file Table_3.DOCX]

**IDI guide for Parents of Sick Children**

Date of interview: [___|___ /___|___/ 2 | 0 | 1 | 9 ]

Time of interview: Start [___|___:___|___] End [___ |___:___|___]

County: __________________________________________________

Sub-county: __________________________________________________

Child’s Age: __________________________________________________

Premature Child: Yes [___] No [___]

**Note:** *Small and sick newborns for this review refers to a newborn who is born preterm, small for gestational age, has an illness, or suffers from a birth complication, and requires hospitalization during the neonatal period.* ***Infant and young children for this discussion refers to children anywhere from birth to 2 years old.***

*Watoto wadogo na watoto wachanga wagonjwa hapa inamaanisha mtoto mchanga aliyezaliwa kabla ya watati wake kufika, mdogo kwa umri wa ishara, ako na ugonjwa ama ana shida tangu kuzaliwa na anahitaji kulazwa hospitalini punde tu baada ya kuzaliwa.* ***Watoto wadogo hapa inahusu watoto kati ya umri wa kuzaliwa hadi miaka miwili.***

**Tell me about yourself**

1. How old are you?

*Uko na umri gani?*

1. How many living children do you have (biological or other)? Were all your biological children born at term?

*Uko na watoto wangapi walio hai (uliowazaa ama wengine)? Je watoto uliowazaa ulijifungua baada ya miezi tisa?*

**Probe; how many are between 0-2 years old, is there any child with chronic illness or requires frequent hospital visits and why**.

***Ulizia****: wangapi wako kati ya umri wa miaka 0-2, je kuna mwenye ako ana ugonjwa sugu ama anauhitaji wa kutembelea hospitali mara kwa mara na kwa nini?*

1. Tell us about child health services at this (Site Name) facility (type of services offered, providers at this facility, time taken to be served)?

*Tafadhali tuambie kuhusu huduma za afya za watoto kwa hiki (jina la kituo) kituo cha afya( aina ya huduma zinazopeanwa, watoa huduma kwa hiki kituo cha afya, mda unaochukua kuhudumiwa)?*

- 1. What do communities feel about child health services at this facility?

*Je, jamii inahisi nini kuhusu huduma za afya za watoto kwa hiki kituo cha afya?*

- 1. At community level what type of services are offered and how frequently (probe: outreaches, home visits, other and who provides them)?

*Kwa kiwango cha jamii, ni aina gani za huduma zinapeanwa na mara nyingi kiasi gani (ulizia huduma za nje, huduma za nyumbani, nyengine na ni nani anazipeana)?*

1. What do you know about referral of sick babies (0-2 years) between hospitals? What normally happens – please explain.

*Je ni nini unajua kuhusu rufaa ya watoto wadogo wagonjwa (miaka 0-2) kati ya hospitali?ni nini hutokea kwa kawaida- tafadhali eleza.*

**Topic I: Experiences of your interactions between you and health care providers.**

***Uzoefu wa maingiliano kati yako na watoa huduma za afya.***

**Now let us focus on the last visit you made for services for young sick child 0-2 years old.**

***Sasa tugeuze mtazamo kwa mara ya mwisho ulitembelea hiki kituo ili kupata huduma za watoto wadogo kati ya miaka 0-2.***

1. How long ago did you last receive services for your newborn (0-28 days), baby (29 days – 60 days), or young child (and child (61 days - 2 years) was sick in this facility?

*Ni lini mara ya mwisho ulipokea huduma za afya wakati mtoto wako mchanga (siku 0-siku 28) ama mtoto wako mdogo(siku 29-siku 60) na mtoto(siku 61- miaka 2) alikuwa mgonjwa kwa hiki kituo cha afya?*

**Probe if it was: inpatient (NICU/NBU)? Pediatric ward services, outpatient, chronic illness, any other?**

***Ulizia: kama ilikuwa mgonjwa wa kulazwa (NICU/NBU)? Wodi ya watoto wadogo, wagonjwa ambao si wakulazwa, ugonjwa sugu, nyengine?***

1. Thinking back to this time, tell us about your experience with the providers when your baby/child was unwell?

*Ukifikiria huo wakati,tuambie jinsi ilivyokuwa na wahudumu wa afya wakati mtoto wako alikuwa mgonjwa?*

**Probe for:**

- ***What departments did you visit?***

*Ulitembelea idara/ vitengo gani?*

- ***Are parents greeted nicely? Did the providers introduce themselves? \***

*Je wazazi husalimiwa vizuri? Je watoa huduma walijitambulisha?*

- ***Did providers explain how to care for your child? Did they explain what needed to be done (e.g. tests) in a timely way that you could understand?***

*Je watoa huduma walieleza jinsi ya utunzaji wa mtoto? Je walieleza ni nini inapaswa kufanywa (eg vipimo) kwa mda unaofaa na kwa namna ungeelewa?*

- ***Were you allowed to asked questions? Were able to ask questions? Were your questions answered satisfactorily?***

*Je uliruhusiwa kuuliza maswali? Je uliweza kuuliza maswali? Je maswali yako yalijibiwa na ukaridhika?*

- ***What types of instructions about follow up care did the provider give after your baby was discharged?***

*Ni aina gani ya maagizo kuhusu utunzaji wa kufuatilia ambayo mhudumu alikupatia baada ya mtoto wako kutolewa?*

- ***How did the providers speak to you - in a friendly tone? Rushed tone?***

*Je wahudumu walikuongelesha vipi- kwa sauti ya kirafiki? Kwa sauti ya hasira?*

1. In your opinion, were there any disagreements or challenging moments with service providers? **Probe:** If yes please describe what happened and where? How were they resolved? How did these challenging instance/s affect you and your child?

*Kwa maoni yako, je kulikuwa na kutokubaliana au matukio ya changamoto na wanaotoa huduma* ***ulizia:*** *kama ndio tafadhali eleza ni nini ilitokea na wapi? Yalisuluhiswa vipi? Je haya matukio ya kutoelewana yaliathiri vipi wewe na mtoto wako?*

- 1. **Did you at any point feel humiliated in anyway while your sick child received care in this facility? Probe: what, how and why**

*Je kuna wakati wowote ambao ulihisi aibu kwa njia yoyote ile/ kudhalilishwa wakati mtoto wako mgonjwa alikuwa akipokea utunzaji kwa hiki kituo cha afya* ***ulizia nini, kivipi na kwanini***

1. While you were in hospital, what kind of help did you receive to care for your sick child? What other help did you want or wish you had received? Please explain.

*Mlipokuwa hospitalini je mlipokea msaada wa aina gani ili kuwasaidia katika utunzaji wa mtoto wenu mgonjwa? Ni msaada upi mwengine mlihitaji ama mlitamani mgepata? Tafadhali eleza.*

**Probe for - emotional support, instructions about childcare, information or counselling?**

***Ulizia:msaada wa kihisia, maagizo kuhusu utunzaji wa mtoto,habari ama ushauri?***

1. ***Did you receive any other support? If yes what type of support?***

*Je ulipata msaada wowote? Kama ndio msaada wa aina gani?*

1. ***Who supported you and the family during that time?***

*Je ni nani alikupatia wewe na familia msaada wakati huo?*

1. ***what support would you require in the long term***

*Ni msaada wa aina gani utahitaji kwa mda refu ujao?*

1. ***What other support did parents like you receive during that time?***

*Ni msaada wa aina gani mwengine ambao wazazi kama wewe walipokea wakati huo?*

1. ***were you ready and or able to follow-up with the instructions/ counselling given by the provider?***

*Je ulikuwa tayari na ama ulikuwa na uwezo wa kufuatilia hayo maagizo ama ushauri uliopewa na huyo mhuduma wa afya?*

**Topic II: Understanding caregiver context for respectful care for newborn and young children**

***Kuelewa mukthada wa walezi kwa ajili ya utunzaji wa watoto wachanga na watoto wadogo wenye heshima***

1. Do you think providers try to build a relationship with parents of the sick young children under the age of 2 years?

*Je unafikiria kwamba wahudumu wanajaribu kujenga mahusiano na wazazi wa watoto wadogo wagonjwa chini ya umri wa miaka miwili?*

- 1. ***How are newborns and sick young children are “treated” by the health providers i.e. do you think providers try to build a relationship with the babies?***

*Ni jinsi gani watoto wachanga na watoto wadogo wagonjwa wanahudumiwa na watoa huduma wa afya.****i.e*** *je unafikiria kwamba wahudumu wanajaribu kujenga uhusiano na watoto*

- 1. ***Do you think providers treat babies as human beings? Please explain.***

*Je unafikiria kwamba wahudumu wanawahudumia watoto kama binadamu (utu)? Tafadhali eleza.*

- 1. ***Do you feel the provider is gentle with the babies? Please explain.***

*Je unafikiria wahudumu ni wapole kwa hao watoto?Tafadhali eleza.*

- 1. ***How do providers speak to parents of sick children?***

*Je wahudumu hao huwaongelesha vipi wazazi wa watoto wagonjwa?*

1. Think of all the different people who might have a role in making sure children 0-2 years are treated in a friendly, sensitive and humane way. What are the roles of: **parents/caregivers, fathers, families, community members, community health workers Community health volunteers (CHVs), healthcare providers, health managers and government county/national?**

*Fikiria kuhusu watu wote tofauti ambao wako na jukumu la kuhakikisha kwamba watoto wa miaka 0-2 wanahudumiwa kwa njia ya kirafiki, inayojali na njia ya utu/kibinadamu. Ni nini majukumu ya:* ***wazazi/ walezi, baba, familia, wana jamii, wahudumu wa afya wa kijiji, wahudumu wa afya wa jamii wa kujitolea, mameneja wa afya na serekali kaunti/ kitaifa?***

- 1. **How are caregivers prevented from caring for their babies? Probe: how is it different for men and women?**

*Ni jinsi ganzi walezi wanazuiwa kutuko kwa utunzaji wa watoto wao?* ***Ulizia:*** *unatofautiana vipi kwa wanaume na wanawake?*

1. Please can you give any example of insensitive or inhumane treatment for newborn and sick young children 0-2 years in a hospital? What does this poor treatment look like at: Maternity, OPD, Pediatric ward Post-natal ward? **Probe in, public, private; faith-based, referral hospitals**

*Tafadhali unaweza peana mfano wowote wa huduma ambazo hazijali au huduma ambazo si za ubinadamu(utu) kwa watoto wachanga na watoto wadogo wagonjwa kati ya miaka 0-2 wakiwa hospitalini?je hizi huduma mbaya zinakaa aje kwa:vyumba vya wamama wajawazito, OPD, wodi ya watoto, wodi ya baada ya kujifungua?* ***ulizia;*** ***kwa hospitali za umma, hospitali za kibinafsi. Hospitali za imani, na/au hospitali za rufaa***

1. At what age are newborns and young children more likely to receive poor quality of care and/or insensitive/inhumane treatment?

*Ni katika umri gani watoto wachanga na watoto wadogo wako na uwezekano zaidi kupokea utunzaji duni na/ au huduma ambazo hazijali/ huduma ambazo si za kibinaamu(utu)?*

- 1. **Probe if differentiated by**: age group: 0-28 days; 29 days – 12 months and; 13 months – 24 months of life?

***Ulizia****: kama imetofautishwa na kikundi cha umri: siku 0-28, siku 29–miezi 12, miezi 13– 24 au maisha?*

1. What do you think might be reasons for poor quality of care, unfriendly, insensitive or inhumane treatment? **Probe for:** facility-related issues (e.g. space/infrastructure, staff, medicines/supplies, any other)

*Ni nini unafikiria inaweza kuwa sababu ya utunzaji duni usio wa kirafiki, usiojali au utunzaji usio na utu/wa kibinadamu?****ulizia****:mambo yanayohusiano na kituo cha afya (kwa mfano nafasi/ miundo mbinu, wafanyi kazi, madawa/ vifaa vya madawa, nyengine)*

- 1. **How does it work for routine outpatient care and inpatient services (e.g. NBU, NICU/pediatric and post-natal ward)?**

Inafanyaje kazi kwa utunzaji wa kawaidia kwa wagonjwa wasiolazwa na huduma za waliolazwa**( eg NBU, NICU/ wodi za watoto na wodi za baada ya kujifungua)?**

1. How does poor quality of care or insensitive/ inhumane treatment of newborns and young children affect caregivers and families of newborn and young children 0-2 years?

***Probe for: Psychologically/emotionally, physically, health seeking behaviour, adherence to follow up instructions, relationship with providers, any other?***

*Je, ni jinsi gani utunzaji duni au utunzaji usiojali / usio wa kibinadamu (utu) wa watoto wachanga na watoto wadogo unaathiri walezi na familia za watoto wachanga na watoto wadogo kati ya miaka 0-2?*

***Ulizia: kisaikologia/ ama kihisia, kimwili, tabia za kutafuta utunzaji wa afya,kufuatilia maagizo ,mahusiano na wahudumu wa afya, nyengine?***

1. How could families be more involved in deciding what and how their newborn and sick young children (0-2 years) can be cared for?

*Je, ni jinsi gani familia zinaweza kuhusishwa zaidi katika maamuzi ya ni nini na ni vipi watoto wao wachanga na watoto wadogo wagonjwa (0-2) wanaweza kupata utunzaji?*

***Probe for while receiving:***

***Ulizia wakati wanapokea:***

- 1. ***Maternity/postnatal ward***

*Wodi ya wamama wajawazito/ wodi ya baada ya kujifungua.*

- 1. ***Inpatient services such as Newborn Unit (NBU), Newborn Intensive care unit (NICU), Pediatric ward***

*huduma za afya za kulazwa kama wodi ya watoto, kitengo cha watot wachanga, kitengo cha utunzaji mkubwa.*

- 1. ***Ambulatory outpatient services for sick young children 0-2 years***

*Huduma za afya zinazopewa watoto wachanga ambao ni wagonjwa kati ya miaka 0-2 na hawalazwi hospitalini.*

1. Do you think parents **should/can** do some of the things that nurses do? (for example tube feeding, turning baby’s position, changing soiled linen, giving some medication, in newborn and pediatric service areas for children 0- 2 years – for example the neonatal unit/NBU? If yes Probe for:

*Je unafikiria kwamba wazazi wanafaa/ wanaweza kufanya mambo mengine ambayo wahudumu wa afya wanafanya (kwa mfano kuwalisha kupitia kwa bomba, kugeuza sehemu ya watoto, kugeuza pamba/ nguo zilizochafuka, kupeana madawa, kwa maeneo ya huduma ya watoto wachanga na watoto wadogo kati ya miaka 0-2- kwa mfano NBU? Kama ndio* ***ulizia:***

- 1. ***Which ones would you be happy to do yourself? Have you ever done them?***

*Ni zipi ungefurahia kufanya kibinafsi? Ushawai zifanya?*

- 1. ***Would you be interested in being even more involved? Why or why not?***

*Je ungependelea kushughulishwa zaidi? Kwa nini au kwa nini sivyo?*

1. What are ways to improve relationships between health providers and families of small sick babies and young children?

*Ni njia zipi za kuboresha mahusiano kati ya wahudumu wa afya na familia za watoto wadogo wagonjwa na watoto wadogo?*

**Anything else to add?**

*Jambo lengine la kuongezea?*
