# Supplementary material for: “You can't even ask a question about your child”: Examining experiences of parents or caregivers during hospitalization of their sick young children in Kenya: A qualitative study
Source: Front Health Serv. 2022 Oct 3;2:947334. doi: 10.3389/frhs.2022.947334 (PMC10012665; doi:10.3389/frhs.2022.947334)
Supplement: Supplementary file 4 [file Table_4.DOCX]

**Structured observational tool to document how sick young infants and children up to two years are managed in hospitals in Kenya**

| **Name of facility being observed** |  |
| --- | --- |
| **Name of persons making observations** |  |
| **Date** |  |
| **Department being observed** |  |
| **Observation Started (time)** |  |
| **Observation completed (time)** |  |
| **Document number of children seen that day in that department** |  |
| **Number of nurses in the department being observed today** |  |
| **Number of other providers (clinical officer, nutritionist , doctor)** |  |

**Introduction**

This tool allows us to observe what happens during care provided to newborn or sick children up to two years. In this process, we aim to document the following:

1. How providers plan day to day work to provide care for children
2. Document how they execute their work in places where children are managed
3. How providers interact with caregivers of children
4. How providers interact with other providers
5. How parents/caregivers and families are treated during hospitalization of their children
6. How parents/caregivers, families and friends interact with each other
7. How caregivers interact with each other during hospitalization
8. Track process of care giving from registration to discharge and planning for follow up care
9. Document all service points and care given in each location (including time a sick baby/child spends from arrival in OPD to admission on pediatric ward)
10. Existing records for caregiving
11. Key indicators that are being collected at point of service
12. Key indicators that are entered in various registers for upward submission

**Each day key in your reflections of the issues above in the tables below and write thick descriptions of the processes. Plan which department area you observe. You will need to sit for a whole day as different things occur at different times**

Postnatal ward/KMC ward/Nutrition corner

Outpatients/MCH and specialized clinics eg CCC TB, ENT Clinics

NICU/NBU/ICU –(for children between 1-2 years)

Pediatric ward

**Part A: How providers plan service delivery and overall service structure for sick children**

| **Key features of interaction: please use the un-bolded questions as prompts for specific types of actions, care, and environment.** | **Describe key observations and detail process and interactions each day [in notebook]** |
| --- | --- |
| **How do providers plan their day to day work as they provide care for sick children? What do they do?** |  |
| 1. Organize equipment for service delivery 2. Organize tools /registers 3. Organize drugs/vaccines for use 4. Organize other elements of service 5. How shifts are organized 6. Duty allocation and workflow management during that shift |  |
| **Document how they do their work in places where children 0-2 years are managed** |  |
| 1. Document start time of service delivery compare this with recommended start time and end time   When do providers take breaks for lunch or tea break. How long are these breaks, do they take time at the same time or different times?  Which hour/s are the busiest in the department? (Detail the number of patients and why you think the hour/s was busy)  How busy are providers? Are they equally busy at all times or are there lulls? |  |
| How do they manage long queues/many patients admitted?  Is there any triage processes?  How do providers work during emergencies of any kind (provide details of how it is managed, promptness, teamwork, communication and by who?) |  |
| Does anyone come to supervise/check on them if yes when and how do they provide supervision  Are providers given time to work on their own uninterrupted -do supervisors interrupt arbitrarily ? |  |
| How do staff go about their jobs- with diligence, calmly, carelessly, distractedly ?  Does staff seem happy, willing, resentful disinterested or afraid? |  |
| **Process of care giving [based on where you are sitting]-describe care process from registration to discharge and planning for follow up care** |  |
| **Care-giving process for newborns** |  |
| 1. How does the facility organize registration process for newborns (0-28) and young children (29 days- 2years)? |  |
| 1. Describe in detail what service is provided in each service point ?(draw the service areas and how patient flow happens) |  |
| 1. How do providers manage observation of children admitted? (consider frequency of medication, request for tests, checking vital signs, other nursing procedures) |  |
| 1. What records/documentation are used in each service point (0-28) and young children (29 days- 2years)? |  |
| 1. Consider how service areas limit positive interaction or enhance interaction with caregivers and families |  |
| 1. Are there other people eg CHW/mentor mothers /kangaroo champion /social workers or other cadre of staff supporting mothers in the department? if there are what are their roles? |  |
| 1. Describe the system of nursing procedures (Timing, who conducts procedures, bed side tests, how it is done, handing and taking over to next team during shifts) |  |
| 1. Describe the system of providing medication of children (Timing of medication, who provides medication and how it is done) |  |
| 1. Clinical review process (Timing , who does it and how it is done- Including test requested during clinical care of children ) |  |
| **How teamwork between providers themselves is exhibited during the period of assessment ?** (detail examples of what you may consider teamwork such as cases of duplication of roles, lapsed communication, nonverbal cues, or if a step in the process of caring for a sick newborn or child is missed. |  |
| **How teamwork between providers and caregivers is exhibited during the period of assessment** (detail examples of what you may consider teamwork eg prompt response to emergencies, timely medication and procedures) |  |

**Part B: Documenting experience of patient centered care**

| **Key features of interaction: please use the un-bolded questions as prompts for specific types of actions, care, and environment.** | **Describe key interactions each day [in notebook]** |
| --- | --- |
| **Observe experiences of mistreatment** | |
| 1. Was there any form of mistreatment that was observed today (physical, verbal, failure to meet clinical standards, stigma and discrimination, Violation of privacy and confidentiality ) 2. If the above occurred, how was it handled? |  |
| 1. Are there any forms of negative behavior that are normalized (eg physical, verbal, failure to meet clinical standards, stigma and discrimination, Violation of privacy and confidentiality. Observe and explain) |  |
| 1. Does the facility environment allow for privacy (Audio or visual)? If yes how? |  |
| 1. Does the facility have any standard operating procedures for children (0-2 years)? if yes detail for each area. ( OPD, Paeds Ward, post-natal ward, NICU and NBU) |  |
| 1. Is there any staff who had recent training on respectful care? If yes, when was it provided, by whom and how have they used the training? |  |
| 1. Does the facility have mechanism of reporting any form of mistreatment when it happens?   Eg are there suggestion boxes? are there conflict resolution mechanism in place? |  |
| 1. If yes, what mechanism exist and how does it function? What forms of mistreatment are reported and to whom? |  |
| **Informed choices about the services** | |
| Do facilities inform caregivers on services being provided? |  |
| If yes, describe how the caregivers are informed on medical procedures being conducted to the young children ( 0-2 years) |  |
| **Describe the consenting process for routine and non-routine/complex procedures** |  |
| **Detail how communication between caregiver and providers happens** |  |
| 1. How do providers explain to caregivers the health and status of their children? |  |
| 1. Do providers give opportunity to caregivers to ask questions? |  |
| 1. Do caregivers ask questions? |  |
| 1. Are providers or caregivers distracted or pulled away during conversations? |  |
| 1. How much time do providers spend with caregivers? |  |
| 1. Do providers use language that is easy to understand and sensitive |  |
| **Protected sleep measures -Safeguarding sleep (Consider the relevant age (0-28 days)** |  |
| Do caregivers get educated on the importance of protected sleep ? If yes how does this happen? |  |
| Consider describing the lighting, air circulation of the room |  |
| Describe general ambience- noisy rooms/environment describing activities- moving trolleys etc |  |
| **Minimizing stress and pain** |  |
| **How do caregivers get involved in managing pain in their newborns or sick children?** |  |
| 1. Are caregivers informed on how to identify pain in children ? if yes provide detail |  |
| 1. Are care givers told how to manage pain? if yes provide details |  |
| 1. Are caregivers told what to do when pain persist? |  |
| **Positioning and Handling (Consider age, time and location of observation)** |  |
| How are caregivers educated on correct positioning of infant for comfort during hospital stay? (Breastfeeding, KMC, sleep) |  |
| How are they educated on handling sick children (eg hygiene, other people handling baby) |  |
| **Optimizing nutrition** |  |
| **How do caregivers get educated on appropriate feeding practices for infants and sick babies? (explain in detail)** |  |
| 1. Are caregivers educated on different feeding methods? (Appropriate feeding methods e.g. exclusive breastfeeding, supplementary feeding and complementary feeding ) |  |
| 1. Are caregivers educated on feeding on demand |  |
| 1. Are they educated on correct positioning and attachment during breastfeeding? -observe if they educate caregivers on breastfeeding difficulties- both maternal and neonatal causes |  |
| 1. For those in NBU/KMC are they educated on feeding using Nasal gastric tube? |  |
| 1. Are they educated to identify children who are not feeding well? (describe details of whether they are educated on what to do if children are not feeding? |  |
| 1. If breastmilk is not available or contradicted describe mechanisms in place to support caregivers on how these children are fed |  |
| **Family partnership, access and involvement** |  |
| **Describe the practice of how family/caregivers are provided access to their infant** |  |
| 1. Are newborns separated from their mothers/caregivers immediately after birth? In what circumstances? Please explain… |  |
| 1. Under what circumstances are families/caregivers given access to the sick young children? (permission to access the ward/area where sick children are ) |  |
| 1. Under what circumstances are families/caregivers not given access to the sick young children? |  |
| 1. How does the facility/providers encourage bonding between caregivers and children during hospital stay (are they allowed to spend time with their babies if yes document how much time is given etc) |  |
| 1. Describe opportunities that parents/caregivers have to participate in their child’s care during hospital care? (how are they included in certain task- giving consent for procedures, feeding, cleaning and changing diapers, turning babies, certain clinical procedures eg feeding using NGT, Physiotherapy) |  |
| 1. Do families have access to resources and support required for managing small sick children during the hospital stay ? (social support eg counselling, waivers, physical-eg hearing aids, wheelchairs, nutritional supplements for malnourished , financial resources, food provided to caregivers) if yes detail how they access the services |  |
| **How are caregivers and families handled during hospitalization of children?** |  |
| 1. Is there a place where they can stay/lodge during the night? is the space adequate |  |
| 1. Are they provided for food and other basic amenities- access to bathrooms , toilet etc |  |
| **Healing environment at hospital** |  |
| Comment on the overall healing environment that promotes safety and sleep for their infants during the hospital stay |  |
| General set up of the ward/NICU/NBU (Baby friendly environment- warmth, colors, play toys etc ) |  |
| **Discharge instructions and follow up** |  |
| Describe whether parents/caregivers are given any instructions/information before they leave the hospital? (e.g. leaflets, medicines, other?) |  |

**Part C: Emotional Support**

| **Focus areas** | **Details of the features/experiences** |
| --- | --- |
| Do providers take any effort to assess the family/caregiver emotional wellbeing? if yes in what ways does that happen? |  |
| Is there any form of supportive care of any kind provided by the hospital to caregivers of children during the hospitalization of the children ? if yes what are the key features of the emotional support system that exist? (KMC support groups, prayer sessions etc) |  |
| How do facilities deal with cases of deaths of children? what form of support exist for such parents? |  |
| Describe any support system that exist for providers working in the ICU/NICU/NBU |  |

**Part D: Documentation of records**

| **Key features of interaction** | **Detail the process** |
| --- | --- |
| What are the existing records for sick young infants and children (What records exist, in what form, how records kept, how do they use records) |  |
| What key indicators are being collected at various service points (document type of data collected, how it is documented for each point of service |  |
| What indicators entered in various registers are summarized in for upward submission |  |
| Are parents/caregivers given a birth notification for newborns? |  |
